# Breadth and Specificity in Pleiotropic Protein Kinase A Activity and Environmental Responses

**DOI:** 10.3389/fcell.2022.803392

**Published:** 2022-02-16

**Authors:** Rachel A. Kocik, Audrey P. Gasch

**Affiliations:** ^1^ Program in Cellular and Molecular Biology, University of Wisconsin-Madison, Madison, WI, United States; ^2^ Laboratory of Genetics, University of Wisconsin-Madison, Madison, WI, United States; ^3^ Center for Genomic Science Innovation, University of Wisconsin-Madison, Madison, WI, United States

**Keywords:** protein kinase A (PKA), spatiotemporal regulation, cAMP microenvironments, environmental response, signaling specificity

## Abstract

Protein Kinase A (PKA) is an essential kinase that is conserved across eukaryotes and plays fundamental roles in a wide range of organismal processes, including growth control, learning and memory, cardiovascular health, and development. PKA mediates these responses through the direct phosphorylation of hundreds of proteins–however, which proteins are phosphorylated can vary widely across cell types and environmental cues, even within the same organism. A major question is how cells enact specificity and precision in PKA activity to mount the proper response, especially during environmental changes in which only a subset of PKA-controlled processes must respond. Research over the years has uncovered multiple strategies that cells use to modulate PKA activity and specificity. This review highlights recent advances in our understanding of PKA signaling control including subcellular targeting, phase separation, feedback control, and standing waves of allosteric regulation. We discuss how the complex inputs and outputs to the PKA network simultaneously pose challenges and solutions in signaling integration and insulation. PKA serves as a model for how the same regulatory factors can serve broad pleiotropic functions but maintain specificity in localized control.

## Introduction

**FIGURE 1:**
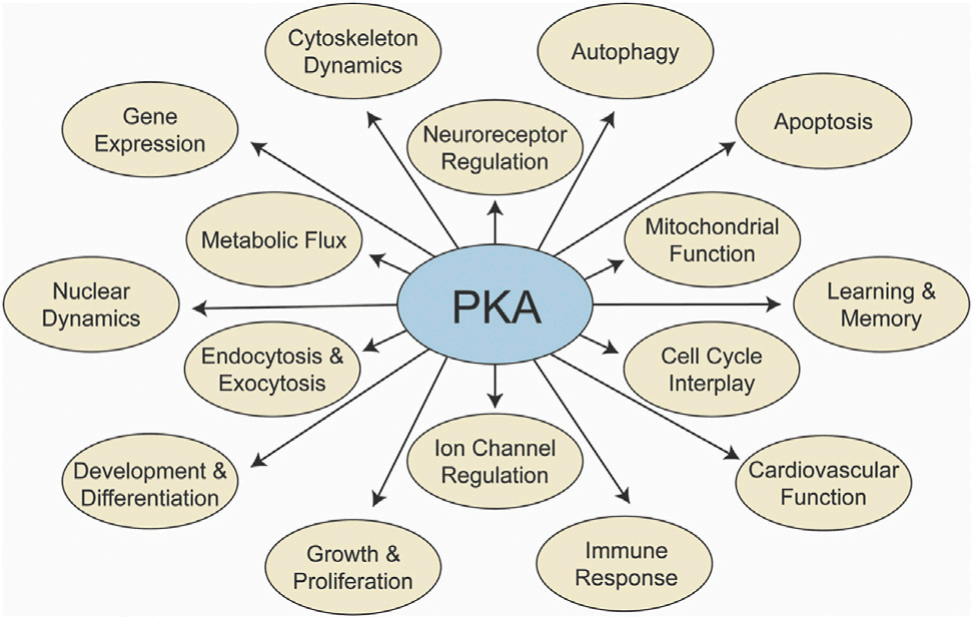
PKA signaling influences many processes. A summary of processes influenced by PKA signaling, through direct phosphorylation of participating proteins.

Protein kinase A (PKA) is among the best studied eukaryotic kinases, owing in part to its essential function in many cellular processes. The kinase can phosphorylate hundreds of proteins, including enzymes linked to metabolism, cellular machinery involved in transcription and translation, structural proteins in the cytoskeleton, and other kinases and regulators that amplify its effects (Budovskaya et al., 2005; Ptacek et al., 2005; Gnad et al., 2009; Hamaguchi et al., 2015; Sugiyama et al., 2019; Niinae et al., 2021). Which proteins are phosphorylated PKA targets varies according to context (Filteau et al., 2015; MacGilvray et al., 2018; Myers et al., 2019; Walker et al., 2019; MacGilvray et al., 2020) so that the collective action of those phosphorylated proteins can mediate higher-order physiological responses. Indeed, PKA is involved in many processes, from growth and development, aging and stress response, cardiovascular health, learning, and more (Figure 1). This breadth of PKA involvement is underscored by the myriad diseases associated with PKA defects (reviewed in Ramms et al., 2021). These include developmental diseases such as neural tube defects (Huang et al., 2002), heart failure and cardiac diseases (reviewed in Liu et al., 2021), and multiple types of cancer (reviewed in Caretta and Mucignat-Caretta, 2011; Hongying Zhang et al., 2020).

A major question is how the PKA kinase can be involved in so many processes yet maintain specificity in coordinating the correct response for the conditions at hand. Part of the solution emerges from the complexities of the broader PKA signaling network. As summarized below, the network includes multiple upstream activation branches, numerous control points modulating allosteric regulation, and the action of combinatorial network interactions that result in modularity. In addition to the complex network of regulatory players, cell also utilize dynamic regulation to control where, when, and how PKA activity is enacted within the cell.

Here we focus on models of PKA signaling insulation and specificity during cellular responses, using examples from budding yeast *Saccharomyces cerevisiae* and mammalian systems. Yeast cells maintain a simplified PKA network with fewer players than mammalian cells, but it remains one of the best characterized systems for understanding signaling control. Comparing and contrasting yeast and mammalian systems underscores several cases in which signaling principles are conserved, even when the regulatory players have evolved. Below, we present an overview of the PKA regulatory network in yeast and mammals before discussing mechanisms by which cells spatially and temporally regulate PKA signaling, including a focus on newly emerging mechanisms of PKA control.

## Key Players in the PKA Pathway

**FIGURE 2:**
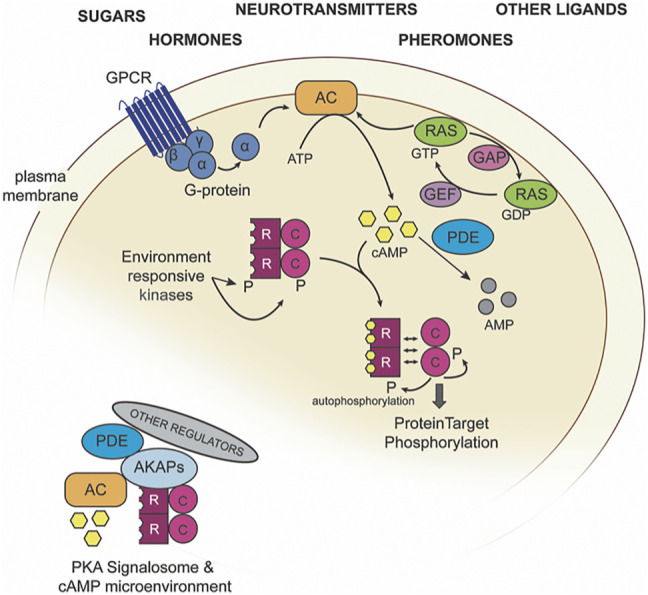
An overview of the PKA regulatory network. PKA catalytic (C) and regulatory (R) subunits can be regulated via cAMP abundance, which is controlled by adenylate cyclase (AC) and cAMP-dependent phosphodiesterases (PDEs). Inset, an example PKA signalosome.

The PKA kinase fits into a broader network with many different levels of control, including multiple input branches and extensive regulatory checkpoints (Figure 2; Table 1). There have been many nice reviews documenting specific regulators in this network (Sassone-Corsi, 2012; Taylor et al., 2013; Conrad et al., 2014). Here we focus on the main points of regulatory control and how breadth and specificity emerge.

**TABLE 1:** PKA subunits and major PKA regulatory proteins. Known PKA players in budding yeast *S. cerevisiae* and mammalian systems. See text for references. + Cyr1 can localize to multiple membranes including in the mitochondria, ER, and plasma membrane (Belotti et al., 2012). * Yeast cells lack proteins orthologs to AKAPs but have other proteins that may serve similar roles, see text for details.

Regulator	Yeast	Mammals
PKA C subunits	Tpk1, Tpk2, Tpk3	Cα (splice variants Cα1, Cα2 and Cα3)
		Cβ (splice variants Cβ1, Cβ2, Cβ3, Cβ4, Cβ3ab Cβ3b, Cβ3abc, Cβ4ab, Cβ4b, and Cβ4abc)
		Cγ
PKA R subunits	Bcy1	RIα
		RIIα
		RIβ
		RIIβ
ACs	Cyr1+	AC1-9 (plasma membrane anchored)
		AC10 (soluble AC)
cAMP PDE Isoforms	Pde1, Pde2	PDE4, PDE7, PDE8 (cAMP specific)
		PDE1, PDE2, PDE3, PDE10, PDE11 (cAMP and cGMP specific)
AKAPs	None*	>50 members (additional splice variants)

### PKA Holoenzyme

PKA is a tetramer of two catalytic (C) subunits and two regulatory (R) subunits. *S. cerevisiae* encodes three paralogous catalytic subunits, Tpk1, Tpk2, and Tpk3, with a single regulatory subunit, Bcy1 (Matsumoto et al., 1982; Toda et al., 1987), while mammalian cells express three main C isoforms that can complex with dimers of the 4 R isoforms (RIα, RIβ, RIIα, and RIIβ). Holoenzyme composition, and also function as discussed in more detail below, can vary; thus mammalian PKA holoenzyme is often described as either Type I or Type II, depending on the regulatory subunits in the complex (RI or RII, respectively) (Scott et al., 1990; Tasken et al., 1993).

While PKA C subunits encompass the catalytic kinase domain, the R subunits serve as a major point of PKA control. R subunits bind the allosteric second messenger cAMP, which induces conformational changes that alter R-C interactions. One long-stranding model is that this conformation change causes R subunit release, allowing C subunits to phosphorylate substrates. Recent evidence in mammalian cells suggests that disassociation of R subunits may be short-lived (Mo et al., 2017; Walker-Gray et al., 2017), and one study proposed that R and C subunits may not even fully release at physiological cAMP levels (Smith et al., 2017). In addition to cAMP-based regulation, R subunits can also be phosphorylated to affect PKA (Manni et al., 2008; Budhwar et al., 2010; Haushalter et al., 2018; Isensee et al., 2018; Søberg and Skålhegg, 2018). For example, in HEK293T cells, RIα phosphorylation alters RIα-C interactions, leading to PKA activation even in the absence of cAMP (Haushalter et al., 2018), while RII phosphorylation in sensory neurons is predicted to disfavor RII-C reassociation for prolonged PKA activation (Ping Zhang et al., 2015; Isensee et al., 2018). Together, differences in holoenzyme composition and post-translational modification can influence PKA activity and provide a substrate for differential control, as discussed more in the subsequent section.

### cAMP Abundance

Since cAMP is a key regulator of R-C interactions, cells sensitively control its abundance. Multiple upstream regulatory branches converge on adenylate cyclase (AC), which catalyzes the conversion of adenosine triphosphate (ATP) to cAMP and pyrophosphate. Yeast harbors a single AC (Matsumoto et al., 1982), while ten isoforms (AC1-10) have been identified in mammalian cells (Table 1). The value of this redundancy is that it allows for differential control, since the different ACs are expressed and localized distinctly across tissue types and can be regulated by disparate upstream signaling pathways (reviewed in Defer et al., 2000; Hanoune et al., 2001; Sadana and Dessauer, 2009; Steegborn, 2014). Along these lines, it was originally thought that AC was membrane associated, but it is now clear that soluble AC can localize to different cellular compartments and respond to intracellular cues (Steegborn, 2014).

In addition to synthesis, cAMP is also controlled through regulated degradation via cAMP-dependent phosphodiesterases (PDEs). Once again, complexity varies from yeast with two PDEs (Uno, et al., 1983; Sass et al., 1986) to mammals with eight different cAMP PDE families (comprising sixteen genes and many different splice variants) (Table 1, reviewed in Azevedo et al., 2014). The diversity in PDE enzymes presents another opportunity for modular control of the PKA network, since PDEs are subject to distinct regulation, intracellular localization, and cAMP affinity (Bender and Beavo, 2006; Hu et al., 2010; Francis et al., 2011; Keravis and Lugnier, 2012; Azevedo et al., 2014). For example, mammalian PDE2 plays a major role in cardiac signaling (Sadek et al., 2020), while PDE10 is implicated in PKA-dependent learning and memory (Rodefer et al., 2005; Giralt et al., 2013; Reneerkens et al., 2013). Like ACs, PDE activity is regulated, notably via phosphorylation by several kinases, including PKA, PKB, PKC, mitogen-activated protein kinases (MAPKs), and Ca^2+^/calmodulin-dependent kinase (Bender and Beavo, 2006; Omori and Kotera, 2007; Keravis and Lugnier, 2012; Azevedo et al., 2014). The large number of mammalian ACs and PDEs provide an opportunity for highly specialized control of cAMP in terms of tissue, environmental response, and upstream control (discussed more below).

### Upstream Regulatory Inputs

Holoenzyme and regulatory points described above serve as hubs for signaling input and integration. Numerous upstream regulatory pathways feed into these hubs to control PKA activity in response to the appropriate cues. Among the best studied are RAS-GTPase and G-protein coupled receptors (GPCRs). In yeast, Ras is one of the primary PKA regulatory inputs regulating growth control, consistent with the classical implication of RAS in human cancers. Ras proteins are stimulated by GTP hydrolysis to activate downstream targets including AC and, therefore, PKA signaling (reviewed in Young et al., 2009; Tamanoi, 2011; Simanshu et al., 2017; Cazzanelli et al., 2018). RAS is itself regulated by GTPase-activating proteins (GAPs) and guanine nucleotide exchange factors (GEFs) as well as many other pathways that modulate activity of those effectors. GPCRs are a large group of transmembrane proteins that receive extracellular signals and facilitate downstream cellular responses (reviewed in Versele et al., 2001; Rosenbaum et al., 2009; Hilger et al., 2018). PKA-activating GPCRs bind distinct ligands, including glucose or other sugars in budding yeast (Colombo et al., 1998; Kraakman et al., 1999) and extending to specific sugars, hormones (like glucagon), and neurotransmitters (such as dopamine) in mammals (Dandan Zhang et al., 2015; Venkatakrishnan et al., 2016; Wu et al., 2017).

One advantage of the many signaling inputs into the PKA network is that PKA can be activated in the context of other cellular responses. But evidence from the literature shows that different modes of PKA activation can produce disparate downstream effects that can be decoupled. Work from our lab showed that up-regulation of PKA is important for anaerobic xylose fermentation in an engineered yeast strain; however, the effects were different depending on how PKA was upregulated. Activating PKA by deletion of the RAS inhibitor *IRA2* enabled rapid fermentation and growth on xylose, whereas deletion of R subunit *BCY1* led to rapid fermentation but arrested growth. Both processes are dependent on PKA, since inhibiting analog-sensitive PKA blocks both processes (Myers et al., 2019). Thus, activating PKA in different ways can lead to different network outputs. It remains unclear if these differences are strictly through PKA activity, or if the different network branches help to coordinate PKA activation with other cellular responses. Nonetheless, the two regulatory branches participate in distinct cellular responses that both require PKA.

### Feedback in PKA Signaling

The importance of proper PKA signaling is also underscored by extensive feedback regulation in the PKA network that can precisely tune PKA activity. PKA can phosphorylate subunits of the PKA tetramer, thereby autoregulating activity (Moore et al., 2002; Manni et al., 2008; Solari et al., 2014; Isensee et al., 2018; Søberg and Skålhegg, 2018). PKA also phosphorylates its upstream regulators in mammals, including ACs, PDEs, RAS, GTPases, and GPCRs (reviewed in Vandamme et al., 2012). In yeast responding to salt stress, phosphoproteomic analysis implicated extensive feedback on PKA-dependent sites of PKA-affecting regulators (MacGilvray et al., 2018). Although the function of each phosphorylation remains to be worked out, feedback in the PKA network is thought to tune the levels and dynamics of signaling through the network (see more below).

In addition to rapid feedback via post-translational mechanisms, slower feedback occurs at the level of gene expression. The induction of genes encoding both PKA activators and repressors is well established in yeast responding to stress: although PKA activity suppresses the stress response, stressed cells induce expression of both positive and negative PKA regulators, including PKA C subunits, R subunit *BCY1*, and phosphodiesterase *PDE2* (Gasch et al., 2000; Segal et al., 2003; Pautasso and Rossi, 2014). The thought is that cells are preparing for eventual re-activation of PKA signaling once cells acclimate. Transcriptional feedback also occurs in mammals: PKA activation of the hallmark transcription factor CREB induces transcription of PDE4, whose accumulation ultimately degrades cAMP to suppress PKA signaling (Swinnen et al., 1989; Vicini and Conti, 1997; D’Sa et al., 2002).

## Specificity and Heterogeneity in PKA Function

The many levels at which PKA activity can be controlled highlights the importance of precise regulation. It also explains how PKA can respond to many different upstream signals and signaling inputs, via the many different pathways and regulatory points that converge on PKA activity. But how do cells mediate disparate outputs of PKA signaling? Long-standing models and new insights from recent studies highlight mechanisms through which cells gain specificity for such a broadly important signaling pathway. Below we summarize several mechanisms that cells use to control the pleiotropic roles of PKA.

### Variation in PKA Holoenzyme Composition and Function

As highlighted above, PKA holoenzyme can vary in composition, thanks to multiple genes encoding PKA C and R subunits, along with alternative splicing in mammals. Several of these isoforms and splice variants are expressed at different abundances in different mammalian cell types, producing tissue-specific holoenzymes. Since these holoenzymes have distinct biophysical properties, compositional differences produce unique signaling effects. For example, in mouse brains, RIβ is more abundant in dendrites, while RIIβ is enriched in axons (Ilouz et al., 2017). Since cAMP-RI has a lower affinity for C subunits than cAMP-bound RII, the difference in holoenzyme composition could alter activation thresholds (Dostmann et al., 1990; Cummings et al., 1996). But this compositional difference also influences PKA substrate specificity: within dendrites, knockdown of RIβ, but not RIIβ, alters PKA-dependent CREB phosphorylation (Ilouz et al., 2017). Holoenzyme differences can mediate these effects through altered protein-protein interactions, subcellular localization, and post-translational regulation from distinct regulatory inputs (Haushalter et al., 2018; Isensee et al., 2018), as discussed in more detail below. Thus, compositional differences in PKA holoenzyme impart specificity in both the reception of upstream cellular cues and the spatial and regional output of PKA-dependent phosphorylation.

### Regulated PKA Localization Changes

One of the simplest ways to alter which targets are phosphorylated by PKA is to change the localization of the PKA tetramer, thereby promoting or restricting access to specific substrates. PKA holoenzyme can be stably localized to the cytosol, nucleus, organelles such as mitochondria and other subcellular compartments, but localization can also change in response to cellular and environmental signals. At least part of this response is dictated by R subunits. In budding yeast, subcellular localization of Bcy1 is controlled by its N-terminal nuclear localization signal and phosphorylation status, wherein phosphorylated Bcy1 is directed to the cytosol and unphosphorylated Bcy1 moves to the nucleus (Griffioen et al., 2001; Griffioen et al., 2003). The resulting relocalization of PKA is indeed dependent on Bcy1, since *BCY1* deletion ablates PKA nuclear translocation in response to low carbon levels (Griffioen et al., 2000). As with Bcy1, the N-terminus of mammalian R subunits can direct regional localization, in conjunction with other interacting proteins that again vary by R-subunit class (Taylor et al., 2012). For example, Protein Kinase Inhibitors (PKIs) that contain their own nuclear export signals can competitively bind C subunits and thus contribute to PKA translocation (Ashby and Walsh, 1972; Gamm and Uhler, 1995; Wen et al., 1995; Herberg et al., 1999). Overall, localization changes can affect PKA activity in two ways: by altering access to the upstream inputs feeding into PKA complexes, and by changing which protein targets PKA encounters by nature of PKA compartmentalization.

### Spatial Tethering and Scaffolding

A more specific case of regulated PKA localization is through tethering or anchoring, where PKA is bound to specific sites in the cell. This is perhaps best understood in mammalian systems, where PKA can be tethered to a variety of locations, including mitochondria, vesicles, lipid membranes, cytoskeleton, and centrosomes. One mechanism of tethering is via direct modification of the PKA holoenzyme. In neuronal cells, myristolization of the holoenzyme tethers it to the plasma membrane to promote interaction with membrane-localized targets (Bastidas et al., 2012; Tillo et al., 2017; Xiong et al., 2021).

PKA tethering can also be specified by protein interactions. Best known are A-kinase anchoring proteins (AKAPs) in mammals that participate in tethering via multi-protein interactions. AKAPs are defined by three domains: a PKA-anchoring domain, a unique cellular localization signal, and domains that organize other proteins to assemble (reviewed in Pidoux and Taskén, 2010; Søberg and Skålhegg, 2018). For example, in HEK293 cells, mitochondrially-localized AKAP Rab32 binds RII-containing holoenzyme to direct PKA-dependent regulation of mitochondrial fission (Alto et al., 2002). Another mitochondrial AKAP, AKAP1, tethers Type II PKA to mitochondria in oocytes, where PKA regulates meiotic arrest and progression through polar body extrusions during oocyte maturation (Newhall et al., 2006). Interestingly, budding yeast lack orthologs of AKAP proteins but express other PKA interactors that may serve the same function. Zds1 and Zds2 are two proteins proposed to function like AKAPs in yeast by directing PKA localization; R subunit Bcy1 has also been proposed to serve this function (Griffioen et al., 2001; Griffioen et al., 2003; Galello et al., 2014).

In addition to simply localizing PKA to specific sites, AKAPs can also scaffold PKA targets to further direct activity. An example is the cardiac-muscle-specific mAKAP that brings together PKA via RIIα along with ryanodine receptor (RyR2), a PKA substrate that mediates calcium efflux and is critical for proper cardiac muscle excitation and contraction (Marx et al., 2000; Kapiloff et al., 2001). Mutant mAKAP unable to bind PKA leads to defective receptor phosphorylation and altered calcium release compared to functional mAKAP (Ruehr et al., 2003). Scaffolding of kinases and their substrates is a common mechanism of directing signaling toward specific sets of targets. In the case of PKA, compositional differences in holoenzyme can lead to a great diversity of scaffolding localizations, based on the distinct protein interactions possible with different holoenzyme subunits.

### cAMP Microenvironments

Beyond bridging PKA with specific targets, AKAPs also bring together PKA regulators including ACs, PDEs, phosphatases, and other kinases to produce highly localized microenvironments known as signalosomes (reviewed in Wong and Scott, 2004; Houslay, 2010; Torres-Quesada et al., 2017). Depending on how regulatory enzymes are coordinated within those microenvironments, they can produce localized regions of high, or low, cAMP abundance that produce second messenger gradients and microcompartments within cells (Bacskai et al., 1993; Zaccolo and Pozzan, 2002; Nikolaev et al., 2006; Lim et al., 2008; Terrin et al., 2012; Maiellaro et al., 2016; Gorshkov et al., 2017).

Microenvironments can also serve as hubs for signaling crosstalk. Cardiac muscle-localized mAKAP described above is a nice example of these principles. In addition to bridging PKA to RyR2, mAKAP colocalizes PKA with PDE4D3, phosphatases PPA2A, MAP kinase ERK5 and its upstream activator MEK5, and calcium-responsive calcineurin (Marx et al., 2000; Dodge et al., 2001; Dodge-Kafka et al., 2005; Pare et al., 2005; Dodge-Kafka and Kapiloff, 2006; Rababa’h et al., 2014). The resulting signalosome enables crosstalk between the cAMP-regulated PKA, ERK5, and calcineurin pathways to regulate cardiac functions (see more below). Failure to integrate these pathways leads to cardiac disease and dysfunction (Pare et al., 2005; Dodge-Kafka and Kapiloff, 2006). Signalosomes can also mediate cross-talk across cellular compartments. For example, Deng et al. (2021) demonstrated that PKA can be localized to autophagosomes *via* interactions between AKAP11 and RIα; upon glucose starvation, AKAP11-mediated autophagic degradation of RIα activates PKA that in turn regulates mitochondrial functions and health (Deng et al., 2021). The great diversity of mammalian AKAP complexes, which differ in their localization, anchoring sites, and protein interactions, can produce a wide range of unique signalosomes, producing highly regulated and specific PKA signaling (Wong and Scott, 2004; Torres-Quesada et al., 2017; Omar and Scott, 2020).

Mechanistic details of how signalosomes function continues to emerge. This area of research has been significantly advanced using optogenetics and fluorescent biosensors. Fluorescence resonance energy transfer (FRET)-based reporters have enabled the quantification of localized cAMP abundance and PKA activity (Zaccolo et al., 2000; Zaccolo and Pozzan, 2002; Nikolaev et al., 2004; Börner et al., 2011; Sample et al., 2012; Chen et al., 2014; Li et al., 2015; Sprenger et al., 2015; Surdo et al., 2017; Shu, 2020; Zhang et al., 2021). FRET occurs when two distinct fluorescence proteins fused to opposite termini of a reporter protein come together upon conformational change, *e.g*., upon cAMP binding or PKA phosphorylation. When fused to specific AKAPs or other proteins, these reporters can inform on the responsiveness of specific PKA signalosomes (Surdo et al., 2017). Other fluorescence-based reporters have also been used, including a recent GFP reporter engineered to phase-separate into distinct foci upon PKA activation (Zhang et al., 2018; Roebroek et al., 2021; Zhang et al., 2021). These tools have been used recently to uncover a wealth of information, from models of cAMP binding and diffusion to oscillatory dynamics of PKA stimulation upon GPCR activation (Zhang et al., 2018; Bock et al., 2020). Several studies have combined sensors with optogenetic approaches to locally modulate the activity of PKA or other signaling proteins and then study the effects (Ryu et al., 2010; Stierl et al., 2011; Sample et al., 2012; Raffelberg et al., 2013; Klausen et al., 2019), including direct activation of PKA at the plasma membrane, mitochondrion, or endosomes (O’Banion et al., 2018; Tsvetanova et al., 2021). One elegant recent example by Truong et al. (2021) used an optogenetics system to activate PKA in different cellular regions to show that PKA localized to cilia, but not the cytosol, inhibits hedgehog signaling in both zebrafish somites and a mouse cell line (Truong et al., 2021). Underscoring mechanisms highlighted in this review, PKA is tethered in cilia *via* its interactions with upstream regulator GPCR Gpr161; feedback interactions between PKA and Gpr161 produce fine-tuned control of hedgehog signaling, enabling proper development (Bachman et al., 2016; Tschaikner et al., 2020; Tschaikner et al., 2021).

### Phase Separation

An under-explored mechanism of PKA control just beginning to emerge is phase separation. Under the appropriate conditions, some proteins can form biomolecular condensates, also known as membraneless organelles. Kinases can phase separate into these condensates in a way that affects their activity. For example, in yeast experiencing nutrient starvation or heat shock, PKA subunits can be sequestered in phase-separated granules including P-bodies (PB) and stress granules (SG) (Tudisca et al., 2010). These granules comprise RNA-modulating enzymes including decay factors or translational regulators, for PB and SG, respectively, and mRNAs that tend to be translationally repressed (reviewed in Luo et al., 2018; Escalante and Gasch, 2021). There is some evidence that PKA is involved in the formation of PB and SG during stress (Ramachandran et al., 2011), and it is possible that active PKA is localized there, as seen for other kinases (Emperador-Melero et al., 2021). Supporting these ideas, yeast PKA C subunits Tpk2 and Tpk3 localize to granules after heat shock, and this localization requires Tpk2 (but not Tpk3) catalytic activity (Barraza et al., 2017). An alternative hypothesis for phase separation is that it protects the kinase from degradation (Ramachandran et al., 2011; Shah et al., 2013). Evidence in yeast shows that stress-induced granules are preferentially delivered to new daughter cells, perhaps providing a mechanism of inheritance of stored material (Garmendia-Torres et al., 2014).

While less well studied, PKA can phase separate in mammalian cells as well. A recent study by Jason Z. Zhang et al. (2020) used a series of PKA and cAMP biosensors to show that RIα subunits in HEK293T cells can phase separate upon cAMP binding, elevating PKA-C activity within granules. The authors showed that RIα phase separation is the driving mechanism for cAMP nanodomains in these cells. Loss of RIα biomolecular condensates results in loss of cAMP compartmentalization, and strikingly this loss appears to underlie increased cell proliferation and adhesion changes (Jason Z. Zhang et al., 2020). As phase separation is a mechanism for concentrating or sequestering biomolecules, a broader role in PKA signaling will likely continue to emerge. It will be especially interesting to see if the effect of PKA phase separation is conserved from yeast to mammals, or if phase separation emerges to be yet-another principle for differential control of PKA localization and activity across cell types and environmental responses.

### Regulation by Localized Feedback and Feedforward Signaling

Feedback and feedforward signaling play important roles in signaling dynamics, sensitization, and buffering (Chen and Elowitz, 2021; Jiang and Hao, 2021). In the case of the PKA network, feedback exists at nearly every step of the network and can be distinct within microenvironments. While the effect of this feedback phosphorylation is not always known, in some cases there are clear signaling consequences. mAKAP discussed above provides a compelling example: PKA phosphorylation activates PDE4D3, which would in effect suppress PKA by degrading local cAMP, whereas Erk5 suppresses PDE4D3 in a cAMP-dependent manner. This complex signaling loop produces localized pulses of cAMP that are important for cardiomyocyte physiology (Dodge et al., 2001; Dodge-Kafka et al., 2005).

Interestingly, specific patterns of feedback signaling can in other cases produce propagating waves of second messenger. One example is seen in the formation of propagating waves of cAMP as measured in the slime mold *Dictyostelium discoideum* (Monk and Othmer, 1990; Wessels et al., 1996; Hashimura et al., 2019; Singer et al., 2019). Starvation induces changes in gene expression that trigger the production of proteins that sense, synthesize, and degrade cAMP. Differences in catalytic rates cause waves of cAMP that propagate across cells and serve as chemoattractants that trigger cells to aggregate into a multicellular slug (Hashimura et al., 2019; Singer et al., 2019). There is also evidence that PKA influences Ca^2+^ waves in developing retinal neurons, and these waves in turn feedback to regulate PKA signaling (Dunn et al., 2006; Drumm et al., 2014). While the basis of establishing propagating waves is not entirely understood for PKA signaling, the mechanisms may be similar to other systems. For example, propagating waves of GTP seen during cell division and migration are established by differential rates of feed-forward and feed-back signaling to enzymes that module GTP levels (Bement et al., 2015; Bolado-Carrancio et al., 2020).

## Conclusion and Perspective

The intricate mechanisms that specify PKA activity–including differences in holoenzyme composition, subcellular localization, signalosome organization, cAMP microenvironments, and feedback regulation–provide a model for how PKA can participate in such a breadth of cellular processes and responses yet provoke distinct and precise downstream outputs. Ultimately, much of PKA activity is controlled in a highly localized manner. Thus, thinking of the pathway as generally “on” or “off” in cells is likely inappropriate. In budding yeast PKA suppresses the environmental stress response, leading to the general model that PKA must be globally inhibited during stress to mount defense systems (Gasch, 2003). Indeed, Pincus et al. (2014) showed that globally inhibiting an analog-sensitive PKA, coupled with artificial induction of transcription factor Hac1, closely mimics the transcriptome response activated by the reducing agent DTT. However, although the transcriptional response is recapitulated, growth control is not: PKA inhibition arrests growth, yet cells exposed to DTT continue to divide and grow during an active response (MacGilvray et al., 2020). This suggests that PKA may not be globally inhibited during the DTT response. Consistent with this notion, many known PKA targets and phosphosites remain phosphorylated and even increase during DTT treatment [which is distinct from salt stress, in which phosphorylation of most of these sites dramatically decreases (MacGilvray et al., 2018)]. Together, these observations argue against global inhibition of PKA signaling, even in a simple single-celled system, and instead raise the possibility of localized responses that permit activation of the stress response concurrent with continued growth.

Another important feature giving breadth to PKA responses may be how the kinase recognizes its targets. Whereas many other kinases recognize docking sequences shared among their target proteins (Reményi et al., 2006), PKA’s choice of targets seems to be largely dictated by proteins that interact with the holoenzyme. Thus while PKA does show specificity for basophilic phospho-motifs (Taylor et al., 2004), much of the specificity for its targets comes via its interaction partners. R proteins, AKAPs, and other proteins discussed in this review bridge PKA catalytic subunits with targets, through direct interaction with those protein targets or simply by localizing PKA in their proximity. This modularity enables vast opportunities to specify and even evolve protein targets.

The modularity of PKA signaling presents opportunities and weakness for the cell. On the one hand, the complexity in network organization enables PKA to respond to many different upstream signals and environmental inputs while producing unique cellular outputs. It also imparts systems-level properties, including buffering or sensitizing cells to input signals, producing oscillatory or pulsatile dynamics via feedback loops, and enabling bistability (Chen and Elowitz, 2021). On the other hand, the large number of players in the network increases the mutational targets for disease, potentially providing many routes to unbridled PKA activity. Indeed, mutations in PKA and other regulators in the PKA network are associated with myriad diseases (Huang et al., 2002; Caretta and Mucignat-Caretta, 2011; Hongying Zhang et al., 2020; Liu et al., 2021; Ramms et al., 2021). Continued dissection of the complete PKA signaling network and the systems-level features of different signaling modalities will continue to shed light on this important cellular system.
